# Epitranscriptome of the ventral tegmental area in a deep brain-stimulated chronic unpredictable mild stress mouse model

**DOI:** 10.1515/tnsci-2020-0146

**Published:** 2020-11-03

**Authors:** Nan Song, Jun Du, Yan Gao, Shenglian Yang

**Affiliations:** Center of Military Brain Science, Institute of Military Cognition and Brain Sciences, Academy of Military Medical Sciences (AMMS), The Academy of Military Sciences, No. 27 Taiping Road, Haidian District, Beijing, China, 100850

**Keywords:** depression, deep brain stimulation, nucleus accumbens, chronic unpredictable mild stress, RNA methylation

## Abstract

Deep brain stimulation (DBS) applied to the nucleus accumbens (NAc) alleviates the depressive symptoms of major depressive disorders. We investigated the mechanism of this effect by assessing gene expression and RNA methylation changes in the ventral tegmental area (VTA) following NAc-DBS in a chronic unpredictable mild stress (CUMS) mouse model of depression. Gene expression and *N* ^6^-methyladenosine (m^6^A) levels in the VTA were measured in mice subjected to CUMS and then DBS, and transcriptome-wide m^6^A changes were profiled using immunoprecipitated methylated RNAs with microarrays, prior to gene ontology analysis. The expression levels of genes linked to neurotransmitter receptors, transporters, transcription factors, neuronal activities, synaptic functions, and mitogen-activated protein kinase and dopamine signaling were upregulated in the VTA upon NAc-DBS. Furthermore, m^6^A modifications included both hypermethylation and hypomethylation, and changes were positively correlated with the upregulation of some genes. Moreover, the effects of CUMS on gene expression and m^6^A-mRNA modification were reversed by DBS for some genes. Interestingly, while the expression of certain genes was not changed by DBS, long-term stimulation did alter their m^6^A modifications. NAc-DBS-induced modifications are correlated largely with upregulation but sometimes downregulation of genes in CUMS mice. Our findings improve the current understanding of the molecular mechanisms underlying DBS effects on depression.

## 1 Introduction

Deep brain stimulation (DBS) involves the implantation of electrodes within specific brain regions that provide electrical stimulation to the brain in order to create a therapeutic effect [1]. For example, DBS is effective in relieving the symptoms of movement disorders, most notably Parkinson’s disease and essential tremor [2,3]. In addition, depressive symptoms in patients with treatment-resistant depression can now be alleviated over a period of weeks by nucleus accumbens (NAc)-DBS at a high frequency (145 Hz) [4]; long-term treatment appears to be the important factor in significantly reducing the depressive symptoms of these patients [5,6,7]. Similarly, in a depression mouse model, repeated high-frequency stimulation produces a robust antidepressant effect, whereas acute stimulation is ineffective [8]. These findings suggest that at least some of the behavioral changes induced by NAc-DBS may require changes in the brain that occur over longer periods, such as modulation of neuroplasticity [8].

Chronic unpredictable mild stress (CUMS), a commonly used rodent model of depression, is a condition that induces behavioral changes in mice that resemble the symptoms of major depressive disorders (MDDs) in humans [9]. MDD is an interesting candidate for the treatment with DBS because its pathology involves changes to the regulation of gene expression in various neuronal groups [10,11]. Many of the genes in question support communication between neurons or promote the formation of synaptic structures in neurons [12]. Therefore, it is possible that neural plasticity is important for the long-term effects of DBS. Moreover, it is possible that the long-term use of DBS could correct the behavioral changes observed in MDD, which could be tested using the CUMS mouse model.

Transcriptional changes triggered by an external stimulus are critical for neuronal plasticity [13,14]; these include transcriptional factors encoding the synaptic proteins and signaling molecules that modulate neuronal synaptic properties [13,15]. Thus, changes to epitranscriptomic tagging of mRNAs in the brain play important roles in shaping the neuronal transcriptome in response to stimuli [12]. A previous study reported that changes to the mRNA profile in the ventral tegmental area (VTA) of the brain are relevant to stress-induced depression and resilience [16]. A growing body of evidence indicates that m^6^A (*N* ^6^-methyladenosine)-RNA methylation is also involved in stress-related psychiatric disorders such as depression and anxiety [17,18]. Specific studies have shown that changes to mRNA methylation patterns following a stress response occur in the medial prefrontal cortex and the basolateral and central amygdala of mice and that manipulating m^6^A alters fear memory, transcriptome response, and synaptic plasticity. Thus, stimulus-induced gene expression and protein level changes appear to be correlated with RNA methylation changes in these brain regions [10,18,19], and RNA methylation can be dynamically regulated to influence mouse behaviors [12,18].

In the present study, we aimed to investigate many of the aforementioned phenomena in combination. Specifically, we examined m^6^A-mRNA profiles in the VTA of mice subjected to CUMS-induced depression (a reliable model of MDD [20,21,22]) that were subsequently treated (or not treated) with chronic NAc-DBS while awake and freely moving. Through joint analyses and comparison, we determined the m^6^A-mRNA molecular profiles and signal pathways in the VTA that were related to effects of NAc-DBS on CUMS-induced depression. Thus, our study provides a basis for revealing the molecular mechanisms underlying the complex layer of gene expression regulation that occurs with DBS. By understanding the dysregulation of the m^6^A response, we may begin to understand the pathophysiological processes of psychiatric disorders such as MDD.

## 2 Materials and methods

### 2.1 Deep brain stimulation

Before surgery, 6-week-old C57BL/6J mice were handled for 7 days to habituate them to the experimenter and the stimulation procedure. Subsequently, we anesthetized the mice intraperitoneally (pentobarbital sodium, 0.7%, 10 µL/µg) and, using a stereotaxic frame, implanted electrodes (bipolar, two parallel tungsten wires twisted, 0.22 mm in diameter) reaching the bilateral NAc (coordinates: 1.18 mm anterior, 1.35 mm lateral, and 4.50 mm ventral to bregma), according to the Mouse Brain in Stereotaxic Coordinates (Paxinos and Franklin, 2001; Paxinos and Watson, 2005). Following 5–7 days of recovery and then 2 weeks of a 4-week CUMS regime, we subjected the mice to high-frequency stimulations (100 Hz, 100 µA, 100 µs pulse width) daily for 1 h per day (30 min/unilateral, bilateral stimulation) (i.e., NAc-DBS). Thus, stimulations took place from the second week of CUMS, and they continued for another 2 weeks (i.e., the end of CUMS). We controlled current intensity by a constant-current isolated stimulator and we synchronized our stimulation protocols using Master-8 (A.M.P.I., Israel). We also placed control animals (CUMS-sham) into stimulation chambers daily for 1 h per day and connected them to the stimulator without applying any current. At the end of the experiments, we confirmed the localization of electrodes in the NAc on bright-field photomicrographs of coronal sections. Consequently, mice with misplaced electrodes were excluded from all data analysis. VTA tissue was collected after the 2-week period of stimulation or sham treatment.


**Ethical approval:** The research has been complied with all the relevant national regulations and institutional policies for the care and use of animals. All experiments with animals were performed in accordance with protocols approved by the Institutional Animal Care and Use Committee of the Academy of Military Medical Sciences.

### 2.2 CUMS model

We used a CUMS regimen that followed the procedure originally described by Willner et al. [23] and subsequently adapted for mice [9,24]. This stress model involves the application of repeated, mild physical and psychological stressors. The CUMS paradigm provides a model of a chronic depressive-like state that develops gradually over time in response to stress; hence, CUMS is considered to be a more naturalistic in its induction [20,25]. In our study, we subjected the mice to different kinds of stressors several times a day for 4 weeks in a chronic, inevitable, and unpredictable way. For ethical reasons, the stress procedure did not involve food and water deprivation or immobilization. The stressors used were as follows: damp sawdust; changing the sawdust; placement in an empty cage or an empty cage with water covering the floor; placement in a soiled cage with an aversive odor; cage tilting (45° for 12 h); noise stress (15 min); inversion of the 12:12 h light:dark cycle; lights on or off for a short time during the dark phase or light phase, respectively; foot shock (150 mA, 30 min); and ice-water swimming (15°C, 5 min). We administered these stressors in a pseudo-random manner so that they could occur at any time of day or night and we changed the stressor sequence weekly to ensure the stress procedure was unpredictable. During the behavioral tests, the stress procedure was slightly modified: we reduced the number of stressors applied during the light period so as not to interfere with the tests. In addition, we did not subject test mice to any stressors 12 h before the behavioral tests. Nonstressed mice were left undisturbed in their home cages. In all the experiments, the first 2 weeks of CUMS were DBS-free, whereas the second 2 weeks of CUMS also included administration of DBS. To determine the behavioral effects of the CUMS regimen and DBS treatment and to confirm induction of depression-like behaviors [26,27,28,29,30], we examined sucrose consumption in a sucrose preference test (SPT) and total immobility in a tail suspension test (TST), and forced swimming test (FST) was carried out the next day. We also measured locomotor activity using an open field test (OFT), and no stress was applied the day before the test. We isolated non-stressed animals for 1 day before the OFT in order to match the conditions used for CUMS mice. The methods and results of the above-mentioned behavioral tests are shown in the Supplementary Materials and Supplementary Figures S1 and S2.

### 2.3 m^6^A-mRNA epitranscriptomic microarray detection

We collected the VTA of mice for assessment of m^6^A at designated time points by manual dissection of fresh brains on ice. For each sample, we randomly selected and pooled 6–8 animals from the same group. Brains were immediately flash-frozen after dissection and we then collected defined tissue punches of VTA using a 1 mm round tissue punch while sectioning brains on a cryostat. Total RNA from each sample was quantified using the NanoDrop ND-1000. We performed sample preparation and microarray hybridization based on Arraystar’s standard protocols. Briefly, the total RNA was immunoprecipitated with anti-N^6^-methyadenosine (m^6^A) antibody. We then labeled the modified and unmodified RNAs with Cy5 and Cy3, respectively, as cRNAs in separate reactions using an Arraystar Super RNA Labeling Kit. We combined the cRNAs and hybridized them onto an Arraystar Mouse mRNA Epitranscriptomic Microarray (8 × 60 K, Arraystar; 48,161 mRNAs). After washing the slides, we scanned the arrays in two-color channels using an Agilent Scanner G2505C. We then used Agilent Feature Extraction software (version 11.0.1.1) to analyze the acquired array images. First, we normalized the raw intensities of modified (Cy5-labeled) and unmodified (Cy3-labeled) RNAs using the average log2-scaled spike-in RNA intensities. We then calculated the m^6^A methylation level for the percentage of modification based on the Cy5-labeled and Cy3-labeled normalized intensities. We also calculated expression levels based on the total Cy5-labeled and Cy3-labeled normalized intensities. Subsequently, we identified differentially m^6^A-methylated or expressed mRNAs between the two comparison groups by filtering with a fold change (FC) ≥1.5 and without having replicated samples. In additional analysis, we performed hierarchical clustering using R, gene ontology (GO) analysis using the topGO package (also in R), and pathway analysis using Fisher’s exact test. Details of the experimental process are given in the Supplementary Materials.

## 3 Results

### 3.1 CUMS downregulates gene expression and hypermethylated m^6^A-mRNA modification in the VTA of depression mouse model

**Figure 1:**
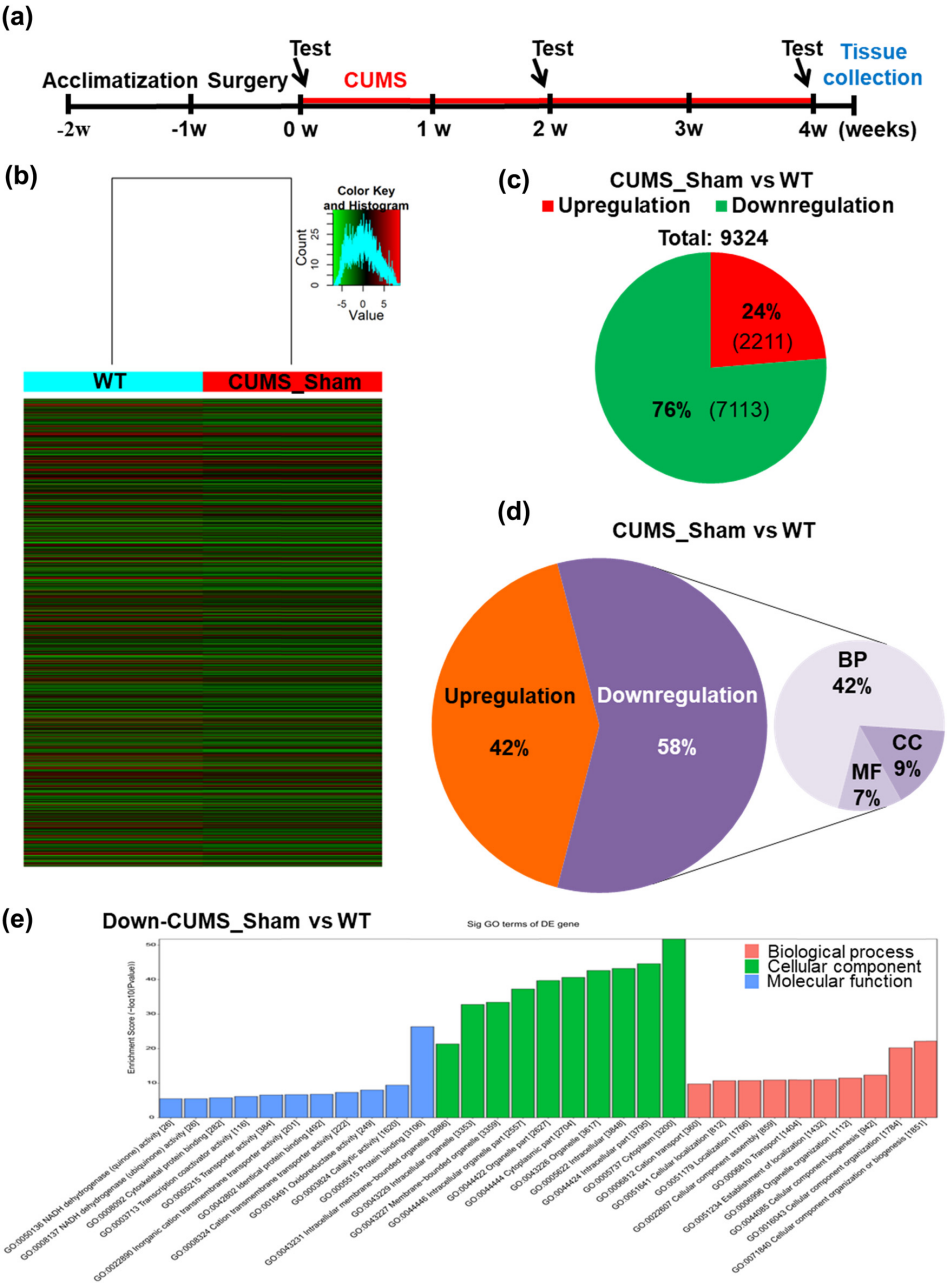
Chronic unpredictable mild stress (CUMS) downregulates gene expression in the ventral tegmental area (VTA) of the depression mouse model. (a) Timeline of the experimental process used in this study. Red lines represent CUMS period. (b) Heatmap showing protein-coding genes for which expression levels changed by at least 1.5-fold in CUMS mice compared with wild-type (WT) control mice. (c) Pie chart representing overall changes in gene expression in CUMS mice compared with WT (upregulation: FC > 1.5; downregulation: FC < 0.67). (d) Gene ontology (GO) analysis of differentially expressed mRNAs in CUMS mice compared with WT (upregulation: FC > 1.5; downregulation: FC < 0.67; BP: biological process; CC: cellular component; MF: molecular function). (e) GO analysis of genes downregulated by at most 0.67-fold in CUMS mice compared with WT.

We examined gene expression and m^6^A-mRNA modification in the VTA, an important nucleus in depression (Figure 1a and Supplementary Figure S3) [31]. We found thousands of gene expression differences between wild-type (WT) and sham-treated CUMS mice (i.e., those that received DBS electrode implants but not electrical stimulation). Compared with WT mice, CUMS induced at least a 1.5-fold increase or decrease in 9,324 genes, with downregulated genes being three times more prevalent than upregulated genes (Figure 1b, c and Supplementary Figure S4a, b and Supplementary Tables S1, S2). By a process of comparison and screening, we found that CUMS-induced downregulation of genes related to gamma-aminobutyric acid (GABA)/dopamine/glutamate/serotonin receptors and transporters (i.e., *Gabrb2*, *Drd2*, *Grin1*, *Htr3a*, *Slc6a18*, *Slc6a2*, *Slc6a20a*, *Slc6a4*, *Slc6a5*, *Slc6a7*, *Slc6a8*, and *Slc6a9*), synaptic and post-synaptic function (i.e., *Snap25*, *Syn1*, *Syn2*, *Syn3*, *Nxph4*, *Ppp1r1a*, and *Dlg4*), and transcription factors (i.e., *Tcf7l2*). In addition, the expression of gene transcripts related to the dopamine signaling pathway (i.e., *Pde1b* and *Syn1*) was also downregulated [17,18,32]. Furthermore, the Fto (EntrezID: 26383) gene, which encodes a nucleic acid demethylase, was downregulated. Given that the inactivation of *Fto* impairs dopamine receptor type 2 (D2R)-dependent control of neuronal activity and behavioral responses [32], the *Drd2* gene, which encodes D2R, was also downregulated in our study. GO analysis of the genes downregulated during CUMS revealed enrichment of the GO terms related to cellular localization, transport, assembly, and biosynthesis (Figure 1d, e and Supplementary Table S3). These data suggest that CUMS, which induced the depressive phenotype in mice, acts through pathophysiological pathways and changes neuroexcitability by altering the expression of key neuronal genes involved in all aspects of neurobiology.

**Figure 2:**
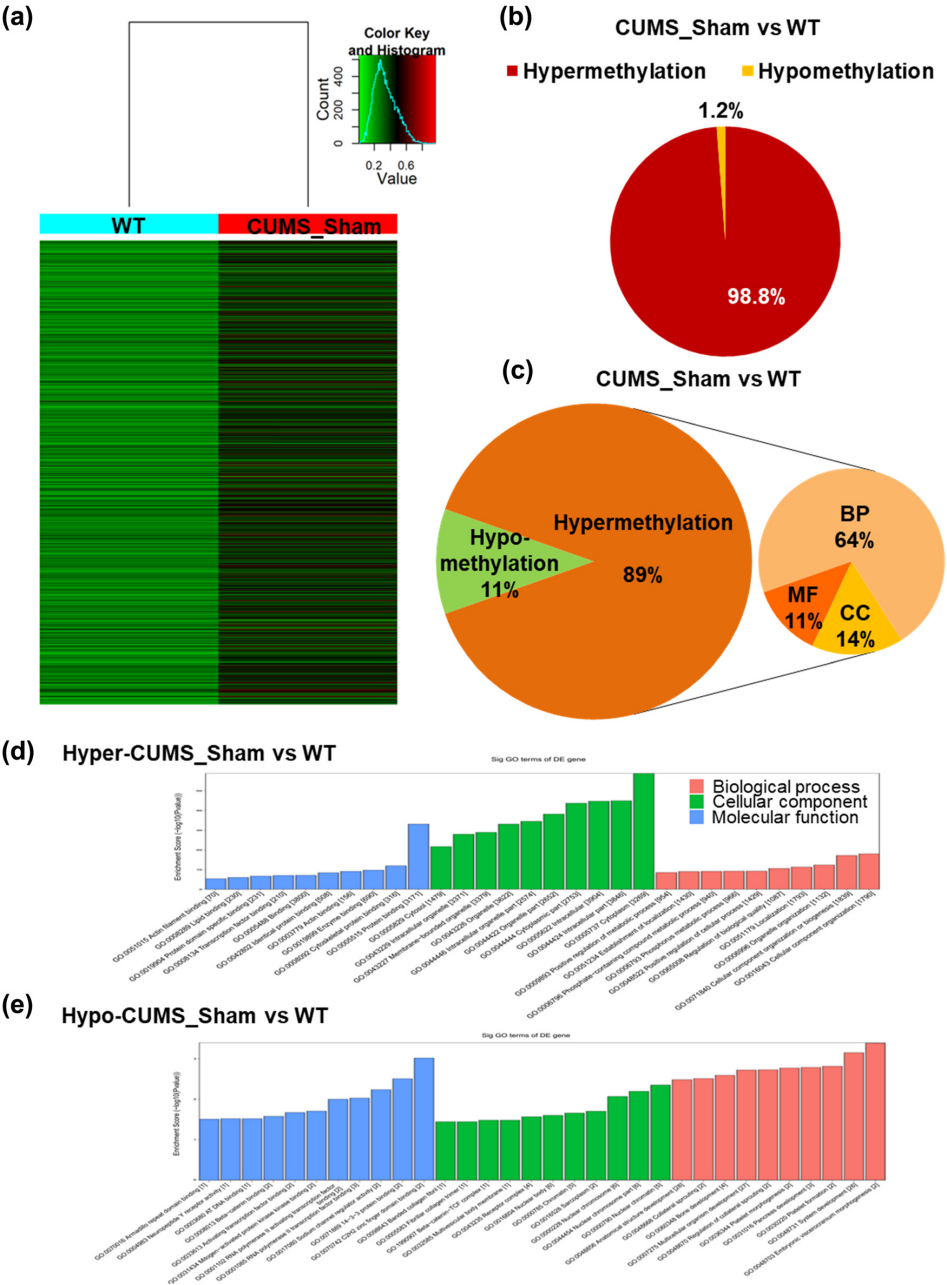
Chronic unpredictable mild stress (CUMS) hypermethylated m^6^A modification in the ventral tegmental area (VTA) of the depression mouse model. (a) Heatmap showing protein-coding genes for which m^6^A-mRNA modification changed by at least 1.5-fold in CUMS mice compared with wild type (WT) control mice. (b) Pie chart representing the overall changes in m^6^A-mRNA modification in CUMS mice compared with WT (hypermethylation: FC > 1.5; hypomethylation: FC < 0.67). (c) Gene ontology (GO) analysis of differentially m^6^A-methylated mRNAs in CUMS mice compared with WT (hypermethylation: FC > 1.5; hypomethylation: FC < 0.67; BP: biological process; CC: cellular component; MF: molecular function). (d) and (e) GO analysis of m^6^A modifications that involved (d) hypermethylation or (e) hypomethylation in CUMS mice compared with WT (hypermethylation: FC > 1.5; hypomethylation: FC < 0.67).

We studied m^6^A-mRNA changes in the VTA of CUMS mice using epitranscriptomic microarray. Compared with WT results, CUMS resulted in 17.8% of genes undergoing m^6^A-mRNA modification (7,605 of 42,655 genes), with ∼98.8% of these genes being hypermethylated (Figure 2a, b and Supplementary Figure S4c, d and Tables S4, S5). These hypermethylation changes induced by CUMS likely lead to changes in the molecular functions of neurons; GO analysis revealed that CUMS-induced m^6^A hypermethylation of genes was associated with the GO terms related to molecular binding function, intracellular organelle/cytoplasmic part, and biosynthesis and metabolic process. In addition, hypomethylated genes were associated with the GO terms related to transcription and translation function, chromatin/chromosome, morphogenesis and development process, and receptor activity (Figure 2c–e and Supplementary Table S3). These results suggest that m^6^A-mRNA methylation in the VTA is relatively high and increases during stress responses.

### 3.2 NAc-DBS upregulates gene expression and hypermethylated m^6^A-mRNA modification in the VTA of CUMS mice

**Figure 3:**
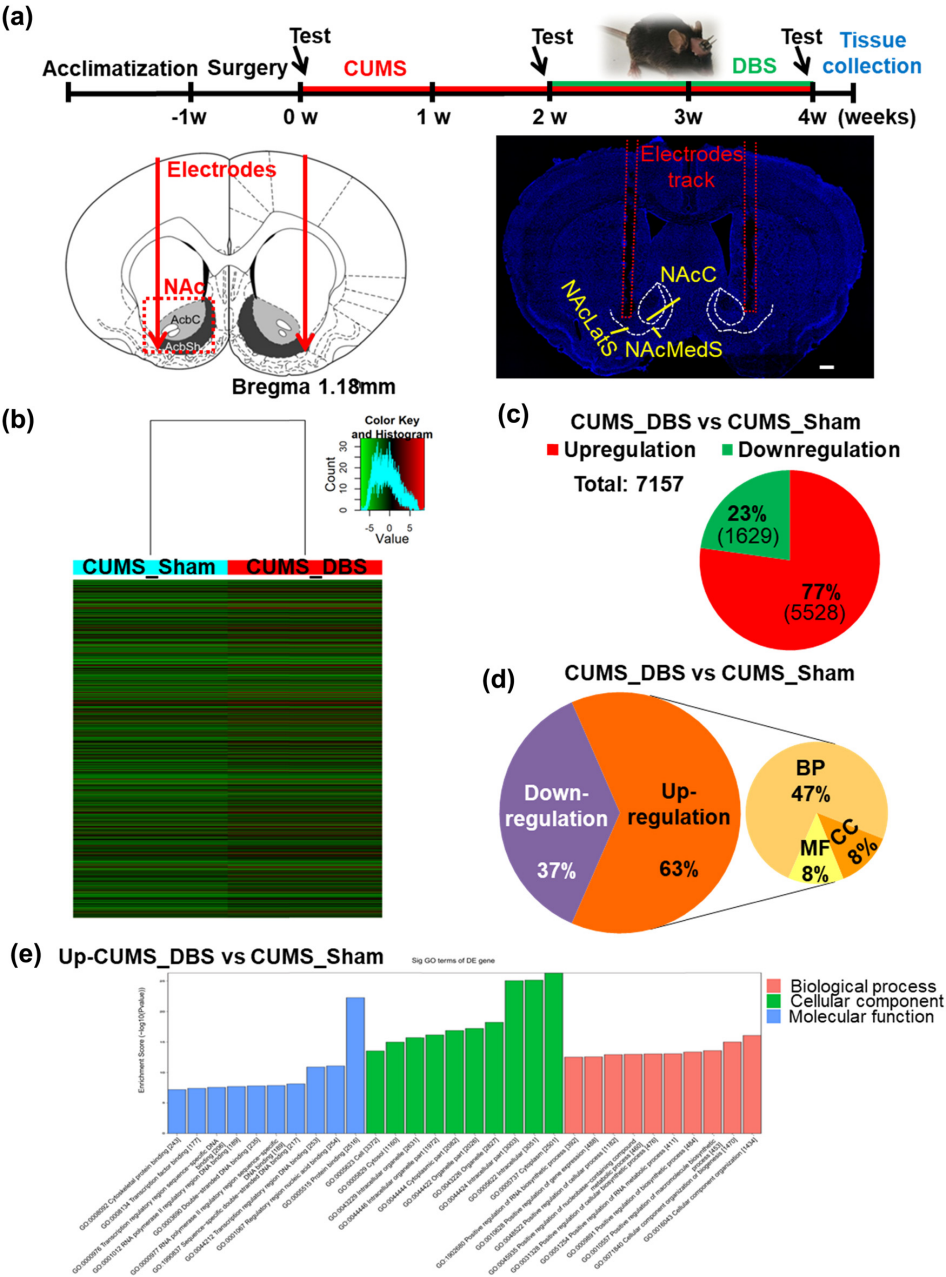
Nucleus accumbens-deep brain stimulation (NAc-DBS) upregulates gene expression in the ventral tegmental area (VTA) of chronic unpredictable mild stress (CUMS) mice. (a) Schematic diagram of electrode placement for DBS and the timeline for implantation and tissue collection (NAc: nucleus accumbens; NAcLatS: lateral shell area of the nucleus accumbens; NAcMedS: medial shell area of the nucleus accumbens; scale bar: 500 µm). (b) Heatmap showing protein-coding genes for which gene expression changed by at least 1.5-fold in CUMS mice subjected to DBS (CUMS_DBS) compared with control CUMS mice that did not receive DBS (CUMS_Sham). (c) Pie chart representing overall changes in gene expression in CUMS_DBS mice compared with CUMS_Sham mice (upregulation: FC > 1.5; downregulation: FC < 0.67). (d) Gene ontology (GO) analysis of differentially expressed mRNAs in CUMS_DBS mice compared with CUMS_Sham mice (upregulation: FC > 1.5; downregulation: FC < 0.67; BP: biological process; CC: cellular component; MF: molecular function). (e) GO analysis of genes that were upregulated by at least 1.5-fold in CUMS_DBS mice compared with CUMS_Sham mice.

With the aim of uncovering the molecular mechanisms underlying the corrective effects of NAc-DBS on depressive behaviors in mice subjected to CUMS, we examined gene expression and m^6^A-mRNA modification profiles in the VTA. Two weeks of high frequency (100 Hz)-dependent bilateral NAc-DBS (30 min/unilateral) caused robust activity-dependent gene expression in the VTA (Figure 3a). Indeed, we found thousands of gene expression differences between CUMS mice that had and had not undergone DBS treatment. Compared with CUMS mice that were not treated with DBS, 7,157 genes were either upregulated or downregulated by at least 1.5-fold following DBS treatment, with upregulated genes three times more prevalent than downregulated genes (Figure 3b, c and Supplementary Tables S1, S2). Comparison and screening showed that DBS induced upregulation of genes related to neuronal activity and synaptic function (i.e., *Adora2a*, *Dnah1*, *Dnah3*, *Dnah14*, *Dnah11*, and *Nxph2*), neurotransmitter receptors and transporters (i.e., *Slc6a5*, *Slc6a7*, *Slc6a8*, *Scl6a18*, *Gabra3*, *Gabra4*, *Gabbr2*, *Gabbr1*, and *Gabrb2*), transcription factors (i.e., Tcf7l2), and mRNA methylation modification (i.e., *Mettl22*, *Fto*, and *Alkbh2*) [17,18,32], which had high frequency-dependent changes in expression in VTA neurons (Supplementary Table S1). Furthermore, DBS upregulated transcripts that encode proteins related to glutamate/neuronal signaling pathways, such as *Grin3b*, as well as those implicated in dopaminergic signaling specifically, including *Pde1b*, *Kcnj6*, *Syn* (synapsin)*1*, *Syn2*, and *Syn3* (Supplementary Table S1). GO analysis of genes upregulated by DBS revealed enrichment in GO terms, relative to CUMS mice untreated by DBS, such as cellular component biosynthetic process, transcriptional regulation process, and gene expression regulation. For molecular function especially, the activities of mitogen-activated protein kinase kinase (MAPKK) binding and mitogen-activated protein kinase (MAPK) binding, which belong to the MAPK signaling pathway and are closely related to neuroplasticity [33], were upregulated (Figure 3d, e and Supplementary Figure S5 and Table S3).

**Figure 4:**
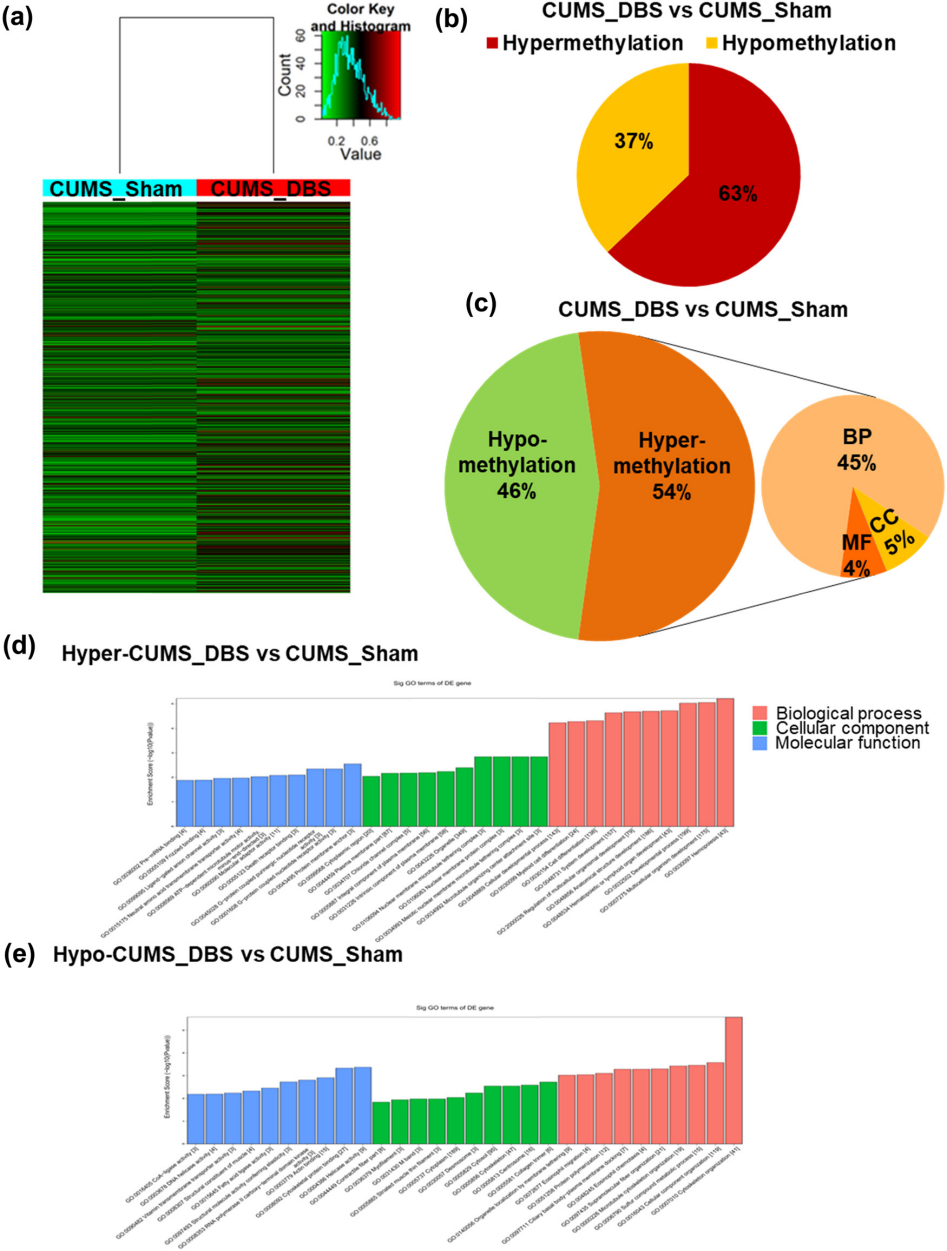
Nucleus accumbens-deep brain stimulation (NAc-DBS) induces hypermethylated m^6^A modifications in the ventral tegmental area (VTA) of chronic unpredictable mild stress (CUMS) mice. (a) Heatmap showing the protein-coding genes for which m^6^A-mRNA modification was changed by at least 1.5-fold in CUMS mice subjected to DBS (CUMS_DBS) compared with control CUMS mice not treated with DBS (CUMS_Sham). (b) Pie chart representing overall changes in m^6^A-mRNA modifications in CUMS_DBS mice compared with CUMS_Sham mice (hypermethylation: FC > 1.5; hypomethylation: FC < 0.67). (c) Gene ontology (GO) analysis of differentially m^6^A-methylated mRNAs in CUMS_DBS mice compared with CUMS_Sham mice (hypermethylation: FC > 1.5; hypomethylation: FC < 0.67; BP: biological process; CC: cellular component; MF: molecular function). (d) and (e) GO analysis of m^6^A modifications involving (d) hypermethylation or (e) hypomethylation in CUMS_DBS mice compared with CUMS_Sham mice (hypermethylation: FC > 1.5; hypomethylation: FC < 0.67).

We also profiled m^6^A-mRNA changes in the VTA of CUMS mice with DBS treatment. Compared with CUMS only induced methylation changes, DBS treatment caused methylation changes in 1,061 genes, 62.9% and 37.1% of which were hypermethylated and hypomethylated, respectively (Figure 4a, b and Supplementary Tables S4, S5). GO analysis revealed that genes with m^6^A hypermethylation induced by DBS were enriched in GO terms related to biological development and differentiation, ion transport and ion channel activity, and protein receptor activity; the hypomethylated genes induced by DBS were related to GO terms such as protein binding/activity, cytoskeleton, and metabolic process (Figure 4c–e and Supplementary Table S3). These findings indicate that m^6^A-mRNA methylation is relatively high in the VTA and increases during DBS treatment.

### 3.3 NAc-DBS dynamically reverses the gene expression and m^6^A-mRNA modification induced by CUMS

**Figure 5:**
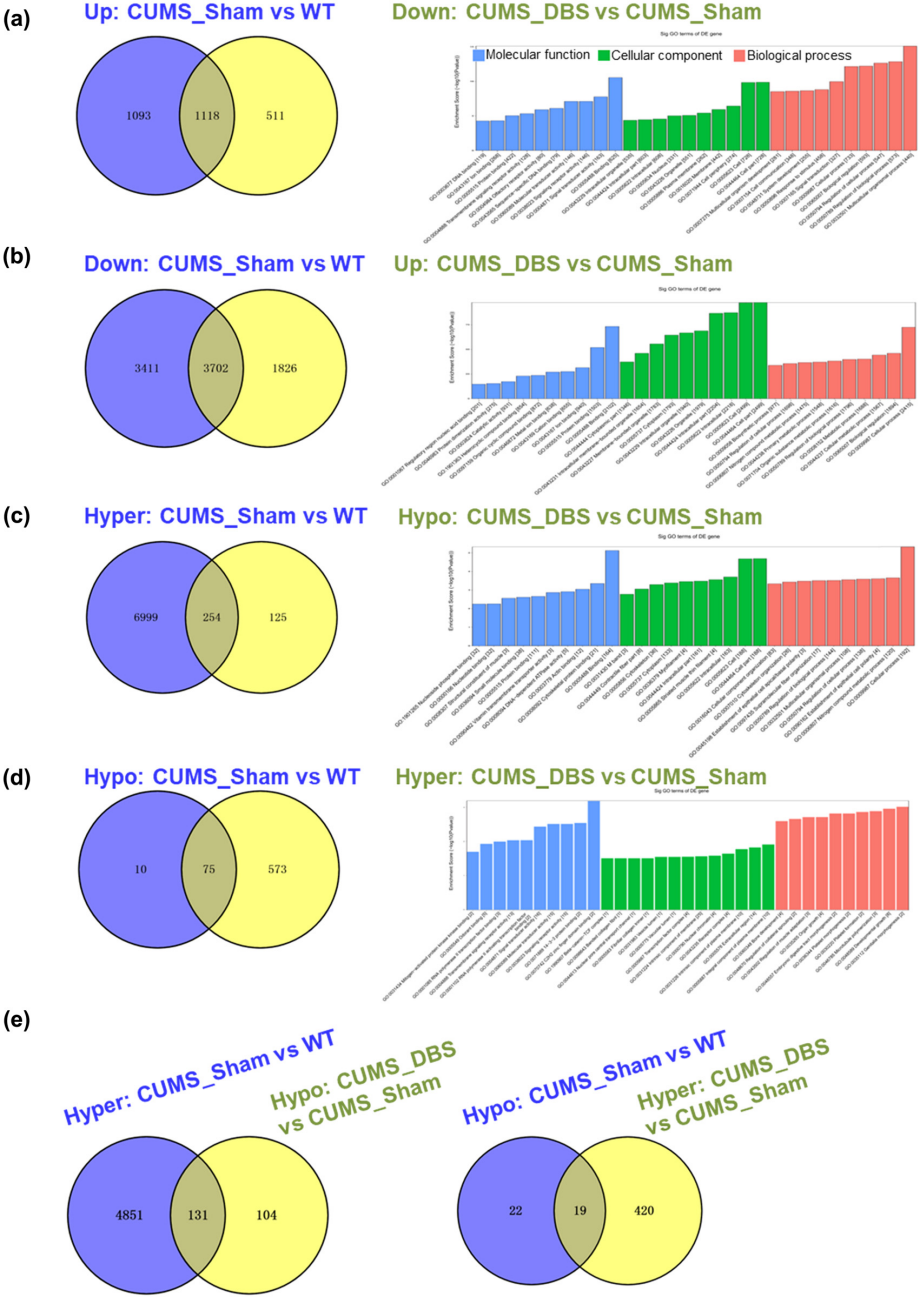
Nucleus accumbens-deep brain stimulation (NAc-DBS) dynamically reverses gene expression and m^6^A-mRNA modification induced by chronic unpredictable mild stress (CUMS). (a–d) Left column: genes can be modulated by CUMS and reversed by DBS at the gene expression and m^6^A modification levels, respectively (up/hyper: FC > 1.5; down/hypo: FC < 0.67); right column: corresponding Go analysis related to data in the left column. (e) Genes with expression levels that do not change substantially in each group (GE: 0.67 < FC < 1.5; GE: gene expression) but for which m^6^A modification is dynamically regulated by DBS (m^6^A-hyper: FC > 1.5; m^6^A-hypo: FC < 0.67).

To understand the potential for DBS to reverse the effects of CUMS, it is necessary to screen for genes that are concurrently modulated by CUMS and DBS at the gene expression and m^6^A modification levels: screening for these genes will further our understanding of CUMS pathogenesis and the mechanism of DBS. At the gene expression level, 1,118 genes that were upregulated by CUMS were downregulated by DBS, while 3,702 genes that were downregulated by CUMS were upregulated by DBS. At the level of m^6^A modification, 254 genes that were hypermethylated during CUMS showed the reverse effect with DBS, while 75 genes that were hypomethylated during CUMS were hypermethylated by DBS. GO analysis of genes that were dynamically regulated by both CUMS and DBS revealed enrichment for the GO terms related to molecular binding function, intracellular part, organism development and biological regulation, biosynthesis and metabolic processes, and response to stimulus. In relation to m^6^A modification, dynamically changed genes were enriched with the GO terms MAPKK binding, transcription factor complex, biological development, and morphogenesis (Figure 5a–d and Supplementary Tables S6, S7).

Interestingly, some genes for which gene expression levels did not change significantly between the sham- and DBS-treated CUMS mice still showed dynamic m^6^A modification regulation with DBS. Specifically, 150 of these genes with m^6^A modification changes were first regulated by CUMS (131 hypermethylated genes and 19 hypomethylated genes) and then regulated in a reverse manner by DBS (Figure 5e and Supplementary Table S6). Given the dynamic genes changes elicited by DBS, the underlying molecular mechanism of the treatment could be explored further at the RNA transcriptomic and epigenomic levels.

### 3.4 Correlation analysis of gene methylation and gene expression in the VTA

**Figure 6:**
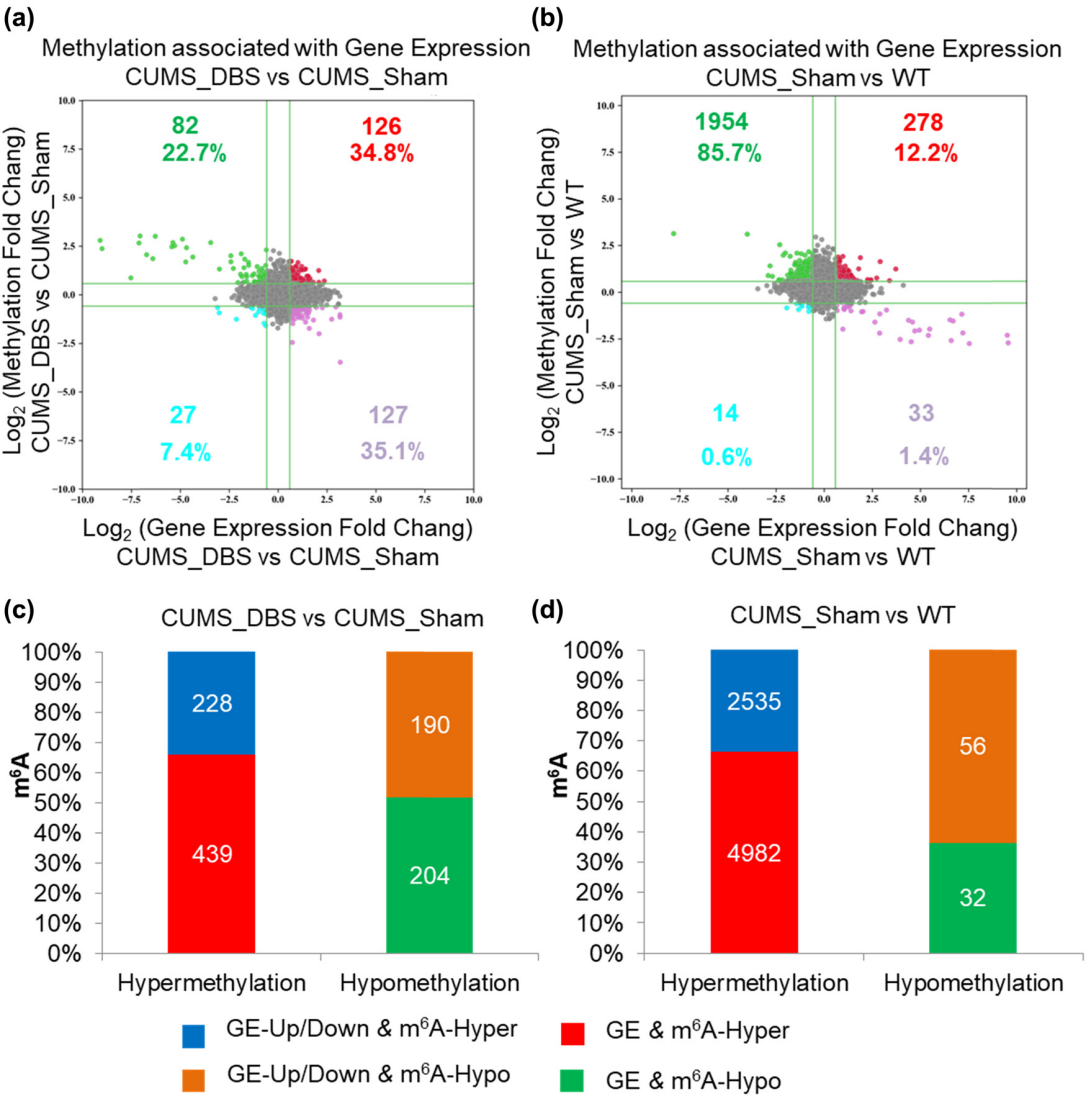
Correlation analysis of gene methylation and gene expression in the ventral tegmental area (VTA). (a and b) Methylation associated with gene expression in (a) chronic unpredictable mild stress (CUMS) mice treated with deep brain stimulation (DBS) and (b) CUMS mice not treated with DBS. Green dots represent hypermethylation genes associated with downregulated gene expression, red dots represent hypermethylation genes associated with upregulated gene expression, blue dots represent hypomethylation genes associated with downregulated gene expression, and purple dots represent hypomethylation genes associated with upregulated gene expression (m^6^A-hyper/GE-up: FC > 1.5; m^6^A-hypo/GE-down: FC < 0.67; GE: gene expression). (c and d) Genes for which the overall expression remained the same but m^6^A changed in (c) CUMS mice treated with DBS and (d) CUMS mice not treated with DBS. The blue column represents the proportion of genes up/downregulated that were hypermethylated (CUMS_DBS vs CUMS_Sham: 228; CUMS_Sham vs WT: 2535), the red column represents the proportion of genes for which overall expression remained the same but that were hypermethylated (CUMS_DBS vs CUMS_Sham: 439; CUMS_Sham vs WT: 4,982), the yellow column represents the proportion of genes up/downregulated that were hypomethylated (CUMS_DBS vs CUMS_Sham: 190; CUMS_Sham vs WT: 56), and the green column represents the proportion of genes for which overall expression remained the same but that were hypomethylated (CUMS_DBS vs CUMS_Sham: 204; CUMS_Sham vs WT: 32) (GE: 0.67 < FC < 1.5; m^6^A-hyper/GE-up: FC > 1.5; m^6^A-hypo/GE-down: FC < 0.67; GE: gene expression).

It has been reported that epigenetic changes, such as m^6^A-mRNA modifications, greatly affect gene expression [34,35,36]. Thus, we performed correlation analysis of gene methylation and gene expression in CUMS mice with or without DBS treatment. We found that among the m^6^A modifications that led to upregulation of gene expression, 34.8 and 35.1% of the genes were modified by hypermethylation and hypomethylation, respectively; conversely, the number of genes downregulated by hypermethylation and hypomethylation was 22.7 and 7.4%, respectively (Figure 6a). In comparison to WT results, CUMS mice that did not receive DBS treatment showed 85.7 and 12.2% downregulation and upregulation of hypermethylated genes, respectively; among the remaining 2% of total genes that were hypomethylated, 0.6 and 1.4% were downregulated and upregulated, respectively (Figure 6b). These results indicate that a linear relationship did not exist between RNA methylation and gene expression in the VTA, and this correlation may depend on different forms of external stimuli (Supplementary Tables S2, S5, and S8).

Gene screening also revealed some genes for which overall expression remained the same but m^6^A changed. Compared with CUMS mice that did not receive DBS, ∼1.0% (439/42,655) and ∼0.48% (204/42,655) of changes involved hypermethylation and hypomethylation in DBS-treated mice (Figure 6c). The hypermethylated genes were related to transcription factors (i.e., *Tcfl5*), neuronal activity, and post-synaptic function (i.e., *Dnah6* and *Fgf23*), neurotransmitter receptors and transporters (i.e., *Gabra5* and *Slc7a9*), and m^6^A methyltransferase and readers (i.e., *Mettl18* and *Hnrnph1*); the hypomethylated genes were related to transcription factors (i.e., *Tfe3*), synaptic function (i.e., *Syt8*), cholinergic receptor (i.e., *Chrna1*), and transporters (i.e., *Slc7a2*) (Figure 6c). In comparison to WT mice, CUMS mice showed ∼11.7% (4,982/42,655) changes involving hypermethylation, with genes that were related to transcription factors (i.e., *Tcf4*), signal transduction (i.e., *Stap2*), neuronal activity and synaptic function (i.e., *Adora1*, *Dnah3*, *Ppp1r8*, and *Snap23*), neurotransmitter receptors and transporters (i.e., *Gabra3*, *Gabrr2*, *Grin2c*, *Grin3b*, *Grm1*, *Htr4*, *Htr7*, *Drd4*, and *Slc6a8*), methyltransferase and demethylases complexes (i.e., *Mettl3*, *Wtap*, *Rbm5*, and *Alkbh4*), and m^6^A readers (i.e., *Hnrnpc*); in contrast, only ∼0.08% (32/42,655) of changes involving hypomethylation was detected in CUMS mice (Figure 6d and Supplementary Table S8). These data suggest that the m^6^A methylation of some genes does not affect their gene expression in the VTA, and this process is also apparently independent of external stimuli.

### 3.5 NAc-DBS alters neural excitability by modulating neuronal signaling pathways

**Figure 7:**
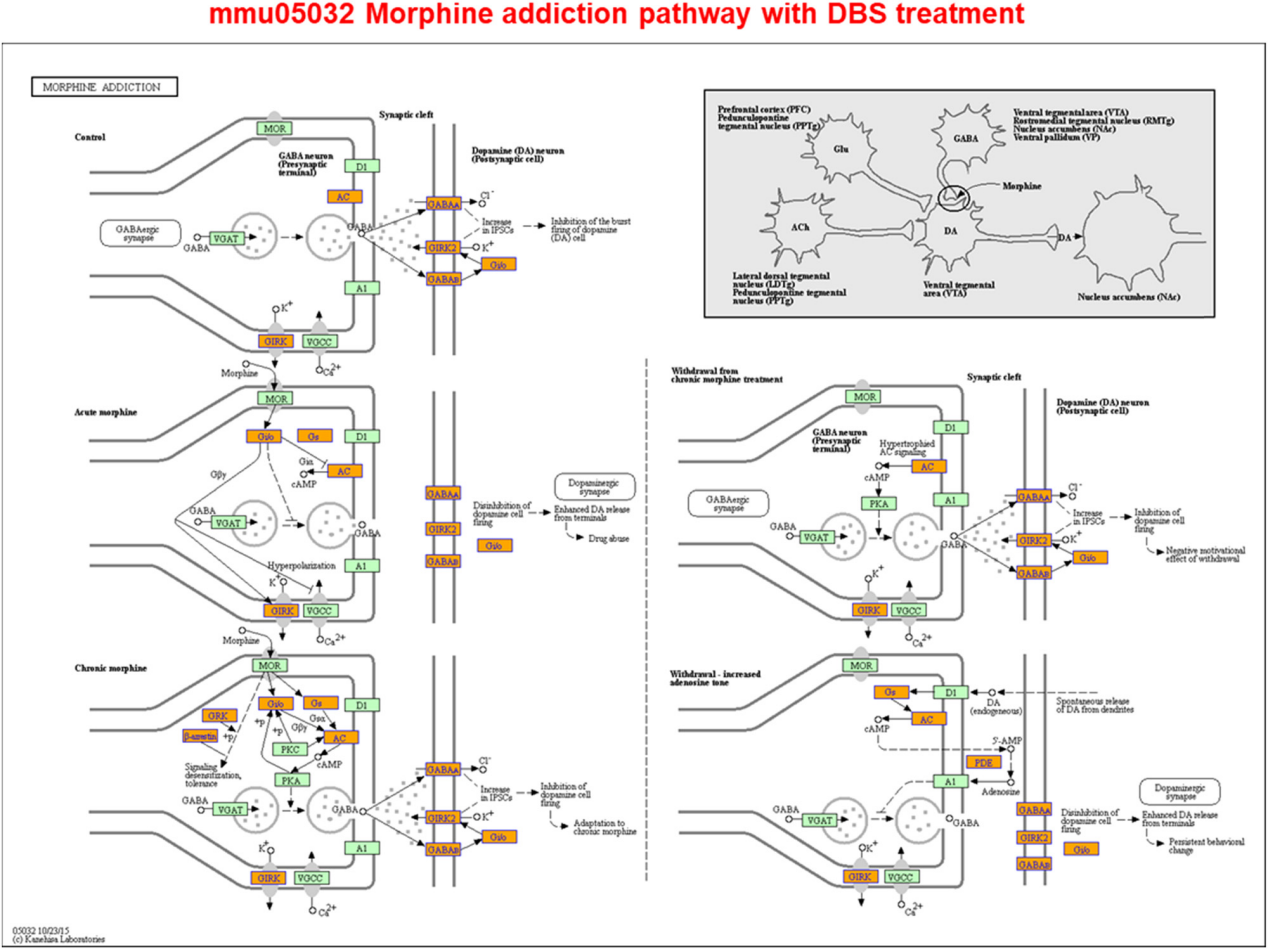
Nucleus accumbens-deep brain stimulation (NAc-DBS) alters neural excitability by modulating neuronal signaling pathways. Screened differentially expressed genes were enriched in the morphine addiction-reference pathway. Orange marked nodes were associated with upregulated genes (FC > 1.5), whereas green nodes had no significance (0.67 < FC < 1.5) (http://www.genome.jp/kegg-bin/show_pathway?mmu05032).

Given the numerous changes to gene expression and m^6^A modification reported earlier, we also determined the related signal pathways that potentially contribute to the long-term effects of DBS. Our results suggested that in the dopaminergic VTA/substantia nigra/caudate putamen/NAc circuitry [37,38,39,40], genes such as *Drd2*, *Drd4*, *Pde1b*, *Kcnj6*, *Syn1*, *Syn2*, and *Syn3* have been implicated in dopaminergic signaling [32], and some of these genes were affected by CUMS at the gene expression level, with these effects being reversed by DBS (Supplementary Tables S1, S4, and S9). However, gene expression in the cocaine addiction-related pathway (i.e., expression of *CREB3L4*, *DLG4*, *GNAI2*, *GPSM1*, *GRIN2C*, *GRM2*, *NFKB1*, and *PPP1R1B* in mmu05030, pathway identifier used in KEGG) was downregulated in CUMS mice treated with DBS (Supplementary Figure S6). Other neuronal signaling pathways, including glutamate signaling (i.e., *Grin1*, *Grin2c*, and *Grin3b*), serotonin signaling (i.e., *Htr4* and *Htr7*), and GABA signaling (i.e., *Gabra3*, *Gabra4*, *Gabbr1*, *Gabbr2*, and *Gabrb2*), were affected by DBS at the gene expression level; all these are involved in the regulation of the excitability of neural circuits. However, at the methylation level, only GABA signaling-related genes were affected (i.e., *Gabra4* and *Gabra5*) (Supplementary Tables S1 and S4). At the gene expression level, GABA signaling was positively correlated with the morphine addiction-related pathway (i.e., expression of *ADCY4*, *ARRB1*, *GABBR1*, *GABBR2*, *GABRA3*, *GABRA4*, *GABRB2*, *GABRG2*, *GNAS*, *GNB3*, *GNG10*, *GNG11*, *GNG13*, *GNG2*, *GNG5*, *GNG7*, *GNG8*, *GRK*, *GRK5*, *KCNJ6*, *KCNJ9*, *PDE10A*, *PDE1A*, *PDE1B*, *PDE4B*, and *PDE7B* in mmu05032, the pathway identifier used in KEGG), whose genes were upregulated by DBS in CUMS mice (Figure 7 and Supplementary Tables S1 and S9).

## 4 Discussion

The intricate dynamics of the neuronal response to stress or stimulation is an important area of research in neuroscience. At present, long-term neuromodulation is emerging as a viable intervention for several neuropsychiatric disorders. We investigated one such neuromodulation technology here—DBS was applied to the NAc to improve depression-like behavior in a CUMS mouse model. Here, we provided a “first look” at the molecular milieu of neurons in the VTA following long-term DBS-induced activation. We believe this is the first study to combine epitranscriptomics and gene expression profiles to address the question of how neuronal activity that is induced by human intervention shapes the function of a neuron.

m^6^A methylation is the most abundant in the mammalian brain and is thought to regulate synaptic plasticity, neurogenesis, axonal growth, learning and memory, and stress responses [41]. The roles played by this newly emerging layer of gene expression control in the central stress response and in stress-related behavior are not yet fully understood [42]; moreover, RNA modifications, along with epigenetic mechanisms, likely represent an, as yet, uncharacterized level of transcriptional regulation that is highly relevant to psychiatry. In analogy to DNA modifications, a diverse set of covalent modifications is present on RNA nucleotides encoding the epitranscriptome, which post-transcriptionally shape gene expression via regulation of RNA stability, translation, and noncoding transcript function [43].

DBS can dynamically correct gene expression and m^6^A-mRNA modification induced by CUMS; this can not only reduce the scope of screening for target genes but also begin to uncover the molecular mechanisms of both depression and the long-term effects of DBS. For example, some genes that were affected at the expression level by CUMS but showed the reversal effect following DBS encode proteins related to inflammation (i.e., *Il13ra2*), stress responses (i.e., *Hspb3*), cell signal transduction (i.e., *Mapk10*, *Gpr171*, and *Card10*), nuclear receptor function (i.e., *Nr2c2*), protein coding (i.e., *Rpl34* and *Sae1*), calcium balance regulation (i.e., *Calr4*), transcription factors (i.e., *Atf3*, *Zfp65* [44], and *Tcf7l2*), neurotransmitter receptors (i.e., *Htr5a*), neuronal activity and synaptic function (i.e., *Dnah11* and *Grm6*), and lipid metabolism (i.e., *Lrp5*). The m^6^A-modified genes in CUMS mice that were corrected by DBS mainly encode proteins related to fatty acid metabolism (i.e., *Slc27a1* and *Acot11*), neurotrophic factors (i.e., *Gfra1*), and transmembrane proteins (i.e., *Tmem168*). In addition, in the case of some key genes, DBS does not affect gene expression but does affect m^6^A modification; these genes encode proteins related to olfactory receptors (i.e., *Olfr*), fatty acid metabolism (i.e., *Ffar1* and *Slc27a1*), and cell signal transduction (i.e., *Crem*). In order to confirm the molecular mechanisms that enable DBS to improve the depressive symptoms shown in CUMS mice, further investigation of the genes listed above, which are directly modulated by DBS, will be required (Supplementary Tables S6 and S7). In particular, the MAPK signaling pathway that were concurrently modulated by CUMS and DBS impacts neural plasticity via modulation of synaptic plasticity, and alterations in MAPK activity are associated with drug abuse as well as neuropsychiatric and movement disorders [33,45]. These results suggested that long-term effects of DBS may affect neural plasticity by regulating MAPK-related pathway.

External stimulations (i.e., CUMS and DBS) affect the epitranscriptome greatly, relative to the effects of gene expression, and mainly promote hypermethylation. For example, a number of genes showed no change in gene expression but changes in m^6^A methylation during CUMS. The genes that were methylated only were closely related to the function and activity of the neural signaling pathway as well as the enzymes involved in the methylation modification process (Supplementary Table S8). Such findings may provide insights into patient populations that do and do not suffer from chronic stress; they also provide evidence that could be the basis for further study into the molecular mechanism of depression at the epitranscriptome level. However, compared with CUMS, only a relatively small proportion of the genes are methylated by DBS. Thus, the external stimulations appear to determine changes in RNA methylation modifications, which subsequently regulate gene expression and finally lead to the changes in the transcriptome profile. Most m^6^A-modified genes were upregulated because of either hypermethylation or hypomethylation after DBS, suggesting the involvement of long-term external stimulation in the correction of the behavioral phenotype mediated by gene expression regulation (Supplementary Figure S7). Future studies are warranted to assess the effects of DBS on the epigenetic modification of disease-related genes. Such additional data will help in refining the patient populations (i.e., those with or without chronic stress) that could benefit from DBS intervention. Although changes in gene expression after DBS treatment (2,626 gene expression changes) in CUMS mice were still slightly different from those in the WT mice (Supplementary Tables S1 and S2), there remained a substantial difference in the epitranscriptomic tagging of RNAs between DBS-treated CUMS mice (9,770 genes with m^6^A modification) and WT mice. In other words, although the behavioral phenotype of CUMS mice is reversed by DBS, the epitranscriptome of these mice differs greatly from that of the WT mice (Supplementary Figure S4c and S4d).

m^6^A modification has been associated with MDD [11]. Loss of motivation and anhedonia in MDD can be associated with abnormalities in the reward system–dopaminergic mesolimbic pathway, in which the NAc and VTA play major roles [46]. The VTA is one of the most important anatomical substrates for drug rewards as well as natural rewards induced by food, sex, and social interactions [47]. Our findings suggest that NAc-DBS has potential beneficial effects on the dopamine signaling pathway. Hess et al. [32] reported that Fto, which encodes a nucleic acid demethylase, is present in dopaminergic neurons and controls cocaine responses. We found that the expression of Fto (EntrezID 26383), which controls the dopamine signaling pathway [32], was downregulated in sham CUMS mice but upregulated in CUMS mice treated with DBS. Another gene that was downregulated by CUMS but upregulated with DBS was *Cartpt* (EntrezID 27220), which encodes cocaine- and amphetamine-regulated transcript (CART) and can be regulated by cocaine and other drugs. Zhang et al. [48] reported that CART is a neuropeptide that is widely expressed by nerve ganglia in brain and neuroendocrine tissues. It is associated with various brain functions, including energy metabolism, feeding/appetite control, stress responses, and neuroprotection [49,50,51]. In the present study, we also found m^6^A changes in *Cartpt*, i.e., decreased methylation levels in CUMS mice treated with DBS. This epigenetic variance in *Cartpt* may affect its dependence on cocaine and the dopamine circuitry function. Dopaminergic signaling governs the control of complex behaviors, and its deregulation has been implicated in various diseases. Cocaine has been demonstrated to exert many of its behavioral effects through modulation of the dopaminergic VTA–NAc circuitry through the dopamine transporter (DAT) [37,38,39,40]. However, in the present study, DBS did not affect the expression and m^6^A modification of DAT-related genes, such as *Slc6a3* [52]. It has been reported that the administration of cocaine results in the robust stimulation of *Fos* mRNA expression in control mice, whereas cocaine-activated *Fos* induction in the VTA/substantia nigra, caudate putamen, and NAc is reduced in *Fto*-deficient mice [32]. Conversely, NAc-DBS could be beneficial in the treatment of cocaine addiction, given that the dopaminergic signaling pathway and cocaine addiction-related pathway are concurrently upregulated and downregulated by DBS. Indeed, DBS could inhibit cocaine addiction by directly modulating the dopaminergic signaling pathway rather than relying on FTO or DAT [40]. It may also depend on the expression level and m^6^A modification changes in *Cartpt*, which is induced by DBS, indicating that DBS does not lead to addiction like cocaine. Furthermore, NAc-DBS can also modulate the morphine addiction-related pathway in the VTA. Previous study has shown that morphine inhibits the activity of GABAergic neurons in the VTA by activating µ-opioid receptors (MOPs) on GABAergic neurons and that VTA GABAergic interneurons are inhibited by morphine, leading to disinhibition of VTA dopaminergic neurons [53]. However, in our study, the gene expression level and methylation level of *Oprm1*, which encodes µ-opioid receptors, were decreased and increased by DBS, respectively (Supplementary Tables S1 and S4). This indicates that in addition to MOPs, CART may also be involved in morphine addiction-related signaling [54,55]. The CART peptide is known to play a role in regulating the behavioral sensitization induced by psychostimulants [56], and the resulting addiction-related anxious and aversive emotions can lead to depression-like behaviors that may ultimately facilitate suicidal actions possibly mediated by GABAergic pathways [57,58]. Therefore, we speculate that the effect of DBS is similar to that of morphine; however, unlike morphine, NAc-DBS does not lead to addiction.

In the VTA, DBS alters the gene expression and m^6^A epitranscriptomic tagging of RNAs that are also pathologically affected in other neuropsychiatric diseases and non-neuropsychiatric disorders. NAc-DBS might prove beneficial in other disorders in which the neural circuits for reward or other neural functions are impaired. Clinical studies have shown that NAc-DBS can not only be used to treat depression but also to treat other neuropsychiatric diseases, such as obsessive-compulsive disorder (OCD), as well as obesity and addiction [59,60]. We were surprised to find that a substantial number of genes and/or their m^6^A modification levels in each of these disorders were induced by DBS in CUMS mice. For example, the gene expression of *Htr4* [61,62,63], *Abca5*/*Abca16* [64,65], *Dusp1*, *Egr2*, *Egr4*, *Csrnp3*, and *Homer1* [66], which are variously related to the above diseases, are all downregulated by DBS (Supplementary Tables S1 and S2). In addition, the m^6^A methylation levels of genes related to these diseases also changed after DBS; such changes occurred with *Slc1a6* (an OCD-associated gene [67,68]: hypermethylation), *Kcnj11* (an obesity-associated gene: hypermethylation) [69,70], and *Dusp1* (an addiction-associated gene: hypermethylation) [66] (Supplementary Tables S4 and S5). We also assessed an overlap of the gene expression and m^6^A modification data with that of other diseases observed in the NAc-DBS dataset. We found that DBS downregulated some genes related to disease signaling pathways, such as those from Alzheimer’s disease (AD) (mmu05010), Huntington disease (HD) (mmu05016), and Parkinson’s disease (PD) (mmu05012), but these genes underwent simultaneous m^6^A hypermethylation. Surprisingly, CUMS itself can also influence the AD and HD pathways at both the gene expression and m^6^A modification levels: related genes are downregulated and m^6^A modification involves hypermethylation (Supplementary Figure S8 and Table S9). Thus, DBS can not only affect many neural circuits but also numerous related genes that intersect with many neuropsychiatric diseases. In addition, DBS has potential beneficial effects on non-neuropsychiatric disease-related signaling pathways, such as obesity, cancers/tumors, leukemia and diabetes pathways (Supplementary Table S9).

Effective treatments for MDD are currently lacking in modern medicine. The vast number of epigenetic changes involved in neuropsychiatric disorders makes it difficult to imagine that a drug-based treatment could ever be effective. As an alternative treatment, DBS is proven to be safe in humans and could offer therapeutic options for otherwise untreatable disorders. Here, we have provided profiles of m^6^A epitranscriptomic tagging genes (RNAs) induced by CUMS or DBS that could provide the basis for subsequent verification studies of gene function but there are still some defects. For example, there was no biological duplication due to the difficulty in sample collection. In future studies, we will make up for the above limitation by improving RNA extraction efficiency. Also, we will test and analyze the gene expression and mRNA profile changes in WT mice receiving only NAc-DBS treatment in future research. According to the literature, DBS of the forniceal can induce gene expression and splicing changes and ultimately promote neurogenesis and plasticity. In addition, approximately one-third of the genes increased by DBS in WT mice overlapped with genes related to many well-characterized immediate early genes, such as *Fos* and *Bdnf*, in addition to numerous transcriptional regulators and signaling components [12].Therefore, the effect of NAc-DBS on gene expression and mRNA profile in WT mice with DBS only is worthy of study. However, with the target genes identified in our study, it may be possible to reveal the pathogenesis of MDD and the effective mechanism of DBS at the neural and molecular level, respectively.
